# Mucinous adenocarcinoma and non-mucinous adenocarcinoma: differing clinicopathological characteristics and computed tomography features in gastric cancer

**DOI:** 10.18632/oncotarget.17389

**Published:** 2017-04-24

**Authors:** Jianxi Zhao, Gang Ren, Rong Cai, Jian Chen, Huali Li, Chen Guo, Wenguang He, Xiangru Wu, Wenjie Zhang

**Affiliations:** ^1^ Department of Radiology, Xinhua Hospital Affiliated to Shanghai Jiaotong University School of Medicine, Shanghai 200092, China; ^2^ Department of Radiotherapy, Ruijin Hospital Affiliated to Shanghai Jiaotong University School of Medicine, Shanghai 200025, China; ^3^ Department of Pathology, Xinhua Hospital Affiliated to Shanghai Jiaotong University School of Medicine, Shanghai 200092, China; ^4^ Department of Surgery, Xinhua Hospital Affiliated to Shanghai Jiaotong University School of Medicine, Shanghai 200092, China

**Keywords:** mucinous gastric carcinoma, non-mucinous gastric carcinoma, clinicopathological characteristics, computed tomography

## Abstract

Mucinous gastric carcinoma (MGC) is a rare histological subtype of gastric cancer. The clinicopathological characteristics and CT features of MGC remain controversial. This study aimed to determine the clinicopathological characteristics and CT features of MGC. We reviewed 62 patients with MGC and 104 patients with non-mucinous gastric carcinoma (NMGC), pathologically confirmed between 2003 and 2015. There are significant differences in some clinicopathological characteristics and CT features between MGC and NMGC. NMGC occurs preferentially in males and more frequently in the lower third of the stomach. Patients with MGC were characterized by larger tumor size, more advanced tumor stages (II and III) and fewer lymphatic invasions. Layered enhancement (83.3%) was the main pattern of MGC, while the most common pattern in NMGC was homogeneous enhancement (52.6%), followed by heterogonous enhancement (34.6%). The degree of enhancement of the inner layer in MGC was significantly higher than in NMGC (ΔCT of portal venous phase: 54.57 Hu vs. 47.19 Hu, P = 0.034), while the middle or outer layer in MGC was significantly less enhanced (ΔCT of portal venous phase: 19.07 Hu vs. 33.09 Hu, P <0.001). Calcifications were more common in MGC (P <0.001). ROC curves revealed that the most effective variables in distinguishing MGC and NMGC were ΔCT of the middle or outer layer in the arterial phase (AUC=0.774) and portal venous phase (AUC=0.774), followed by the attenuation value of the middle or outer layer in the unenhanced phase (AUC=0.763). Calcifications had a high specificity (98.7%) in the diagnosis of MGC. The accuracy (86.1%), sensitivity (83.3%) and specificity (87.2%) of layered enhancement in diagnosing MGC were all high. Therefore, MGC was more likely to have larger tumor size and more advanced tumor stage (II and III) than NMGC. The thicker gastric wall, layered enhancement pattern and calcification were highly suggestive CT features for differentiating MGC from NMGC.

## INTRODUCTION

There are several histological classifications of gastric cancer, such as the Lauren classification [1], Japanese classification [2], WHO classification [3] and so on. According to the World Health Organization classification, gastric carcinomas can be further classified as well differentiated types (well differentiated and moderately differentiated) and poorly differentiated types (poorly differentiated and undifferentiated). Mucinous gastric carcinoma (MGC) is classified as a poorly differentiated type in the WHO classification [3] and an undifferentiated type in the Japanese classification [4]. As a special histological classification, MGC is defined as a gastric adenocarcinoma with a substantial amount of extracellular mucus (over 50% of tumor volume) within the tumors [5]. The evolution of mucinous carcinoma in the stomach has not been completely established. It is generally believed to arise initially as a typical adenocarcinoma that then becomes mucinous with tumor progression. Although studies have reported the distinct clinicopathological features of MGC compared with NMGC, the results remain inconsistent [6–9] due to the variety of the NMGC group, which frequently contains both well-differentiated gastric cancer and poorly differentiated or even undifferentiated gastric cancer [10]. Therefore, we excluded poorly differentiated and undifferentiated carcinomas in the NMGC group.

Mucinous adenocarcinoma is a rare pathological subtype of gastric cancer. To the best of our knowledge, the majority of studies have focused on the clinical characteristics and prognosis of MGC. The radiological findings of MGC are poorly documented. MGC is a term that is typically used on the endoscopic and macroscopic plane; therefore, we aimed to correlate the histological definition of this condition with the realm of imaging. Multidetector Computed Tomography (MDCT) with the stomach fully filled with water is an important method in preoperative evaluation [11]. Other morphology-based imaging tools such as endoscopy, ultrasound, endoscopic ultrasound and MRI have many technical limitations in diagnosis and preoperative evaluation. Therefore, this study elucidates the clinicopathological characteristics and computed tomography features of MGC compared with NMGC to better understand its biological behavior and guide its clinical diagnosis and treatment.

## RESULTS

### Clinicopathological Characteristics

We present the clinicopathological characteristics of the MGC and NMGC patients enrolled in the study in Table 1. Among 62 cases of MGC, there were 41 males (66%) and 21 females (34%) with an age range of 35-80 years old (mean 62 years); meanwhile, among 104 NMGC cases, there were 88 males (85%) and 16 females (15%) with an age range of 33-86 years (mean 64 years). There were significant differences in gender, tumor location, tumor size, macroscopic type, pT staging, pTNM staging, and lymphatic invasion between the patients with MGC and NMGC. MGC and NMGC occurred more frequently in the lower third of the stomach, especially NMGC, with an incidence of 58.7%. MGC was significantly larger than NMGC (6.93 cm vs. 4.11 cm, P<0.001). Early gastric carcinomas of MGC were significantly rarer than NMGC (1.6% vs. 25%). The incidence of stage II-IV in MGC (90.3%) was obviously higher than in NMGC (70.2%). However, lymphatic invasion was more frequent in NMGC (26.0% vs. 9.7%).

**Table 1:** Comparison of clinicopathological characteristics between MGC and NMGC

Clinicopathological variables	MGC	NMGC	P value
	N (%) or mean±SD	N (%) or mean±SD	
Age, years			0.202
<60	27(43.5)	35(33.7)	
≥60	35(56.5)	69(66.3)	
Gender			0.006
Male	41(66.1)	88(84.6)	
Female	21(33.9)	16(15.4)	
Tumor location			0.002
Upper	16(25.8)	29(27.9)	
Middle	22(35.5)	12(11.5)	
Lower	23(37.1)	61(58.7)	
Whole	1(1.6)	2(1.9)	
Tumor size, cm	6.93±4.41	4.11±2.24	<0.001
CEA, ng/ml			0.561
<10	9(18.0)	13(14.3)	
≥10	41(82.0)	78(85.7)	
CA 19-9, U/ml			0.105
<39	16(32.0)	18(19.8)	
≥39	34(68.0)	73(80.2)	
Macroscopic type			<0.001
Type 0	1(1.6)	26(25)	
Type 1	7(11.3)	4(3.8)	
Type 2	35(56.5)	51(49.0)	
Type 3	11(17.7)	18(17.3)	
Type 4	8(12.9)	5(4.8)	
pT staging			0.001
T1	1(1.6)	26(25)	
T2	9(14.5)	14(13.5)	
T3	17(27.4)	18(17.3)	
T4	35(56.5)	46(44.2)	
pN staging			0.441
N0	19(30.6)	44(42.3)	
N1	11(17.7)	17(16.3)	
N2	12(19.4)	19(18.3)	
N3	20(32.3)	24(23.1)	
pM staging			0.542
M0	60(96.8)	97(93.3)	
M1	2(3.2)	7(6.7)	
pTNM staging			0.026
I	6(9.7)	31(29.8)	
II	19(30.6)	26(25.0)	
III	34(54.8)	42(40.4)	
IV	3(4.8)	5(4.8)	
Lymphatic invasion			0.011
Positive	6(9.7)	27(26.0)	
Negative	56(90.3)	77(74.0)	
Venous invasion			0.293
Positive	3(4.8)	1(1.0)	
Negative	59(95.2)	103(99.0)	
Neural invasion			0.224
Positive	5(8.1)	15(14.4)	
Negative	57(91.9)	89(85.6)	

CEA: carcinombryonic antigen

CA 19-9: glycoprotein antigens 19-9

SD: standard deviation

### CT Features

**Figure 1:**
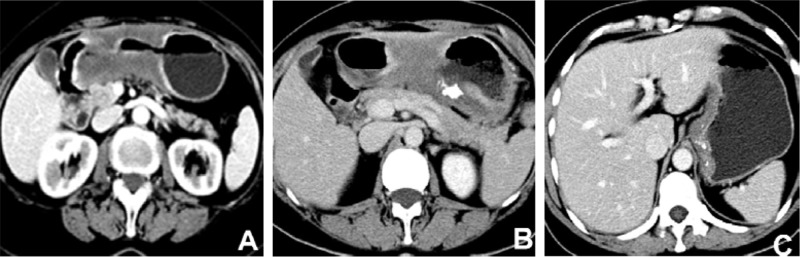
Contrast-enhanced computed tomography images of mucinous gastric carcinoma (**A**) A 67-year-old female with the lesion in gastric antrum showed layered enhancement and centrally hypodense area which was so called “mucin pool”. (**B**) A 60-year-old female with gastric antral cancer showed layered enhancement with irregular calcifications in the “mucin pool”. (**C**) A 49-year-old female with cardiac carcinoma showed layered enhancement and numerous punctate calcifications.

**Figure 2:**
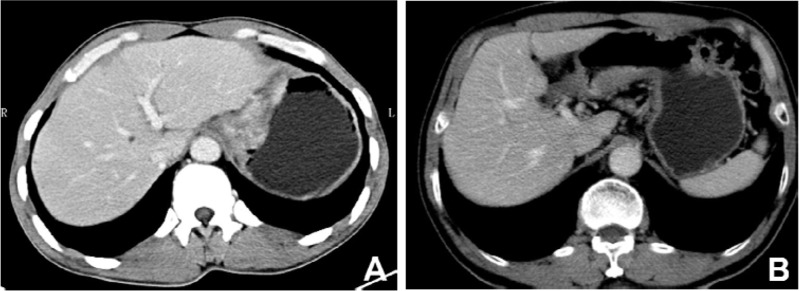
Contrast-enhanced computed tomography images of mucinous gastric carcinoma (**A**) A 40-year-old male with cardiac carcinoma showed obviously heterogeneous enhancement. (**B**) A 73-year-old male with the lesion in gastric antrum showed obviously homogenous enhancement without calcifications.

**Figure 3:**
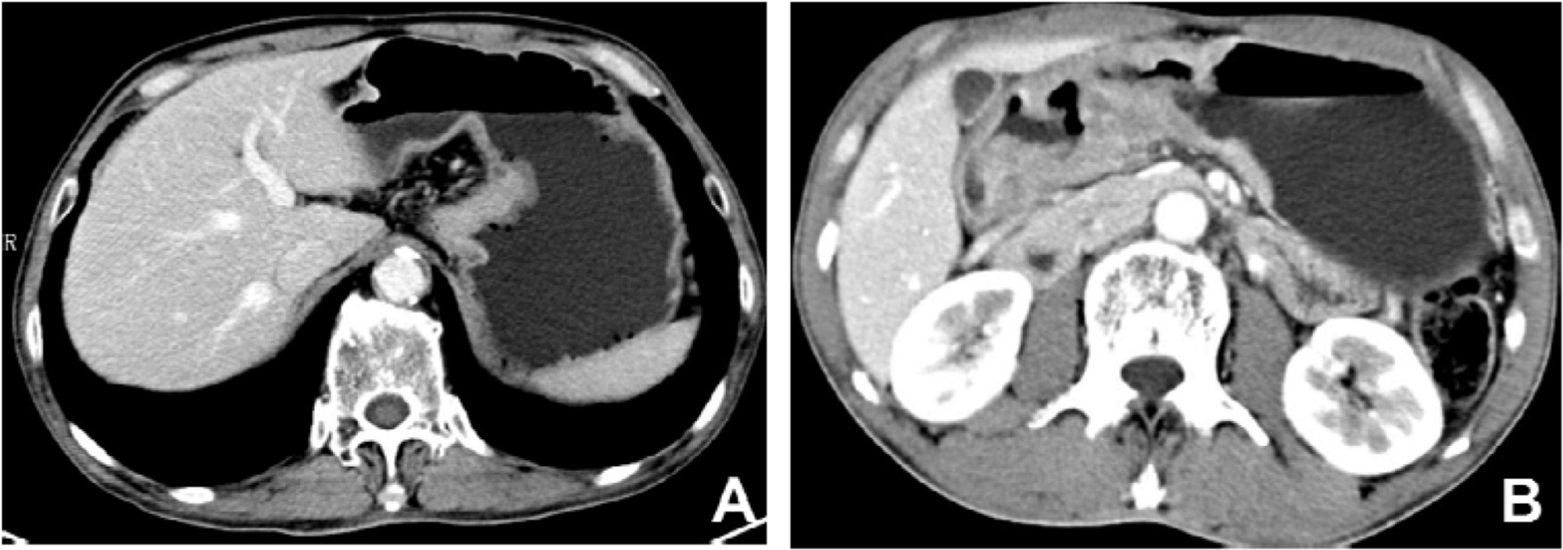
Contrast-enhanced computed tomography images of non-mucinous gastric carcinoma (**A**) A 67-year-old male with cardiac carcinoma showed obviously homogenous enhancement without calcification. (**B**) A 56-year-old male with lesion in gastric antrum showed obviously heterogeneous enhancement without calcification.

Among all cases, there were 30 patients with advanced MGC and 78 patients with advanced NMGC. Their CT features are compared in Table 2. There were significant differences in wall thickness, attenuation value (HU) of the inner-layer in the unenhanced phase, attenuation value (HU) of the middle or outer layer in the unenhanced phase, ΔCT of the middle or outer layer in the arterial phase, portal venous phase and equilibrium phase, enhancement pattern and calcification between MGC and NMGC. The wall thickness of MGC (1.75±0.52 cm) was significantly greater than for NMGC (1.35±0.37 cm). The most common enhancement pattern in MGC was the layered type (83.3%), whereas the most common enhancement pattern in NMGC was the homogeneous type (52.6%), followed by the heterogeneous type (34.6%) (Figures 1, 3). Both homogenous enhancement and heterogeneous enhancement in MGC were very rare (Figure 2). Compared with NMGC, lesions of MGC were more enhanced in the inner layer (portal venous phase ΔCT: 55 Hu vs. 47 Hu, P=0.034) and less enhanced in the middle or outer layer (portal venous phase ΔCT: 19 Hu vs. 33 Hu, P <0.001) (Figures 1–3). Among 30 MGC patients, 10 patients (33.3%) showed punctate, flake or irregular calcifications in tumors (Figure 1). All the calcifications occurred in the middle or outer layer. However, only one case of NMGC showed a suspicious punctate calcification.

**Table 2:** Comparison of CT features between advanced MGC and NMGC

Variables of CT features	MGC	NMGC	P value
	N (%) or mean±SD	N (%) or mean±SD	
Wall thickness, cm	1.75±0.52	1.35±0.37	<0.001
Upper	1.68±0.36	1.38±0.39	0.072
Middle	1.50±0.51	1.36±0.41	0.535
Lower	1.84±0.45	1.30±0.35	<0.001
Whole	3.10	1.93±0.05	0.033
Attenuation value of the inner layer, HU			
Unenhanced phase	34.93±6.75	40.77±7.28	<0.001
Arterial phase(ΔCT)	17.70±14.90	23.19±14.27	0.080
Portal venous phase(ΔCT)	54.57±18.27	47.19±14.98	0.034
Equilibrium phase(ΔCT)	45.06±18.26	41.58±16.25	0.487
Attenuation value of the middle or outer layer, HU			
Unenhanced phase	25.97±5.88	33.26±8.44	<0.001
Arterial phase(ΔCT)	7.00±6.10	15.92±11.31	<0.001
Portal venous phase(ΔCT)	19.07±10.05	33.09±16.13	<0.001
Equilibrium phase(ΔCT)	18.72±9.50	30.55±15.96	0.002
Enhancement pattern			<0.001
Homogeneous	2(6.7)	41(52.6)	
Heterogeneous	3(10.0)	27(34.6)	
Layered	25(83.3)	10(12.8)	
Degree of enhancement			1.000
mild enhancement	4(13.3)	10(12.8)	
Obvious enhancement	26(86.7)	68(87.2)	
Calcification			<0.001
Positive	10(33.3)	1(1.3)	
Negative	20(66.7)	77(98.7)	
Surface dimple or ulcer			1.000
Positive	26(86.7)	66(84.6)	
Negative	4(13.3)	12(15.4)	

Multivariable analysis was performed on the relationship between clinicopathological characteristics and computed tomography features in patients with MGC. The findings revealed close relationships between tumor location, pT staging and enhancement pattern (P = 0.012; P = 0.040, respectively) (Table 3). Six cases of layered enhancement and 1 case of heterogeneous enhancement were observed in upper third gastric cancers; 5 cases of layered enhancement, 1 case of heterogeneous enhancement and 2 cases of homogenous enhancement were observed in middle third gastric cancers; and the 14 patients with lower third gastric cancers all showed layered enhancement. One case of linitis plastica showed heterogeneous enhancement. Four pT2 cases showed layered enhancement; 4 pT3 cases showed 1 case of homogenous enhancement, 2 cases of heterogeneous enhancement and 1 case of layered enhancement; and 22 pT4 cases showed 1 case of homogenous enhancement, 1 case of heterogeneous enhancement and 20 cases of layered enhancement.

**Table 3:** Correlation between clinicopathological characteristics and CT features in MGC

	Wall thickness		P value	Enhancement pattern			P value	Calcification		P value
	≤2cm	>2cm		Homogeneous	Heterogeneous	layered		Yes	No	
Age, years			1.000				0.602			0.442
<60	10	4		0	2	12		6	8	
≥60	11	5		2	1	13		4	12	
Tumor location			0.299				0.012			0.275
Upper	5	2		0	1	6		4	3	
Middle	7	1		2	1	5		1	7	
Lower	9	5		0	0	14		5	9	
Whole	0	1		0	1	0		0	1	
Macroscopic type			0.617				0.198			0.939
Type 1	1	1		1	0	1		1	1	
Type 2	11	5		1	1	15		6	10	
Type 3	6	1		0	2	4		2	5	
Type 4	3	2		0	0	5		1	4	
pT staging			0.809				0.040			0.335
T2	3	1		0	0	4		2	2	
T3	2	2		1	2	1		0	4	
T4	16	6		1	1	20		8	14	
pN staging			0.333				0.123			0.675
N0	9	1		1	0	9		2	8	
N1	3	2		0	2	3		2	3	
N2	2	2		1	0	3		1	3	
N3	7	4		0	1	10		5	6	
pM staging			0.300				1.000			0.333
M0	21	8		2	3	24		9	20	
M1	0	1		0	0	1		1	0	
pTNM staging			0.168				1.000			0.274
I	3	0		0	0	4		1	2	
II	7	3		1	1	6		2	7	
III	11	4		1	2	13		5	11	
IV	0	2		0	0	2		2	0	
lymphatic invasion			0.287				0.287			1.000
Positive	4	0		1	0	3		1	3	
Negative	17	9		1	3	22		9	17	
Venous invasion			1.000				1.000			0.333
Positive	1	0		0	0	1		1	0	
Negative	20	9		2	3	24		9	20	
Neural invasion			0.563				1.000			1.000
Positive	2	2		0	0	4		1	3	
Negative	19	7		2	3	21		9	17	

### ROC Curves

**Figure 4:**
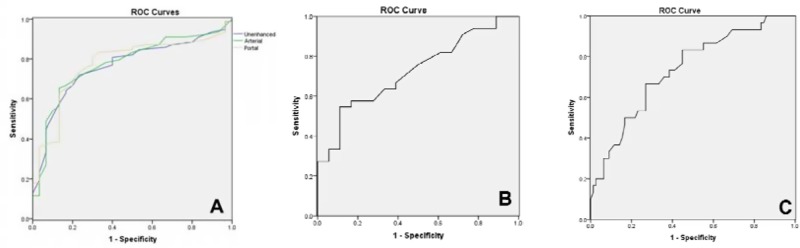
ROC curves of MDCT features (**A**) Unenhanced: Attenuation value of the middle or outer layer in unenhanced phase; arterial: ΔCT of the middle or outer layer in arterial phase; portal: ΔCT of the middle or outer layer in portal venous phase. (**B**) ROC curve of ΔCT of the middle or outer layer in equilibrium phase. (**C**) ROC curve of thickness.

We used ROC curves to evaluate the efficiency of differential diagnosis between MGC and NMGC by lesion wall thickness, attenuation value of the middle or outer layer in the unenhanced phase, and ΔCT of the middle or outer layer in the arterial phase, portal venous phase and equilibrium phase (Figure 4 and Table 4). The results revealed that the most effective variables in distinguishing MGC and NMGC were ΔCT of the middle or outer layer in the arterial phase (AUC=0.774) and portal venous phase (AUC=0.774), followed by the attenuation value of the middle or outer layer in the unenhanced phase and the wall thickness (AUC=0.763; AUC=0.734).

**Table 4:** Parameters of ROC curves

Parameters	AUC	Optimal threshold	Standard error	95% CI	
				Upper bound	Lower bound
Wall thickness	0.734	1.53	0.053	0.838	0.630
Attenuation value of the middle or outer layer in unenhanced phase	0.765	28.5	0.048	0.859	0.671
ΔCT of the middle or outer layer in arterial phase	0.774	11.5	0.048	0.868	0.679
ΔCT of the middle or outer layer in portal venous phase	0.774	19.5	0.048	0.869	0.679
ΔCT of the middle or outer layer in equilibrium phase	0.724	28.5	0.071	0.863	0.585

ROC: receiver operating characteristic curve

AUC: area under the curve

CI: confidence interval

The effectiveness evaluation of differential diagnosis between MGC and NMGC by layered enhancement and calcification is shown in Table 5. The diagnostic accuracy, sensitivity and specificity of layered enhancement were high (86.1%, 83.3% and 87.2%, respectively). Calcification had high accuracy (80.6%) and specificity (98.7%) but low sensitivity (33.3%).

**Table 5:** The diagnostic efficiency of MGC by layered enhancement and calcification

	Accuracy (%)	Sensitivity (%)	Specificity (%)	P value
Layered enhancement	86.1	83.3	87.2	<0.001
Calcification	80.6	33.3	98.7	<0.001
Layered enhancement and calcification	81.5	33.3	100	<0.001
Layered enhancement or calcification	85.2	83.3	85.9	<0.001

## DISCUSSION

Although the incidence and mortality of gastric cancer have gradually decreased in many countries [12], it remains an important cause of death worldwide [11, 13]. Histological classification is an important factor affecting the prognosis of gastric cancer [14], but it is not an independent prognostic factor [15,16]. The incidence of MGC reportedly varies from 2.4% to 10% [4, 16–19]. As a rare histological type, MGC has the special characteristic of secreting extracellular mucus [10, 20]. Studies focusing on MGC have been few due to its particularity and rarity, especially radiographic studies.

Currently, the diagnosis of MGC mainly depends on endoscopic biopsy. However, diagnosis with gastroscopy has certain limitations in the evaluation of infiltration depth, peripheral invasion, lymph node metastasis and distant metastasis. Some reports [11] have shown that the sensitivity of endoscopic biopsy in the diagnosis of MGC is very low. MDCT can definitely show the attenuation value of the primary lesion, infiltration depth, involvement of the surrounding structure and enhancement patterns with little influence from breathing, heartbeat and gastrointestinal peristalsis. Moreover, the MDCT scan can sensitively show punctate calcifications. Therefore, MDCT is the main examination for the preoperative assessment of gastric carcinoma [12]. However, other morphology-based imaging tools have some deterministic technical limitations. For example, MRI cannot overcome the influence of gastrointestinal peristalsis because of the longtime of examination [12]. Due to the limitations of ultrasonic attenuation, EUS cannot clearly display the infiltration depth of advanced gastric cancer [12] and accurately assess the lymph node metastasis and distant metastasis. The diagnostic accuracy of MGC by PET-CT is very low, so PET-CT is worthless in the initial diagnosis and preoperative evaluation of MGC [11].

At present, the clinicopathological characteristics of MGC remain controversial [17]. Studies have shown that MGC occurs mostly in older males [17]. The male/female ratio is 2:1 in the current research. In our study, compared to NMGC, most MGC patients are in the advanced stage and few patients in the early stage at the time of the initial diagnosis, resulting in the larger tumor size, which is consistent with most previous studies [4, 11, 13, 17]. This finding could also explain why the prognosis of MGC is poorer than for NMGC [15]. In terms of macroscopic types, the ulcerative type (type 2) is the most frequent type in both MGC and NMGC. However, there were significantly fewer type 0 cases in MGC than in NMGC. Our results indicate no significant difference in lymph node metastasis between MGC and NMGC, which is consistent with Kim et al [14].

The wall thickness of MGC was significantly thicker than for NMGC, which is consistent with the reports by Yan et al [11]. This difference may be related to the frequently advanced stage of MGC. The enhancement patterns of gastric cancer are divided into homogeneous enhancement, heterogeneous enhancement and layered enhancement. Most of the enhancement patterns of MGC were layered enhancement characterized by a high-low or high-low-high intensive mode on enhanced CT images. The degree of enhancement of the inner layer in MGC was significantly higher than in NMGC. However, there is currently no report of the inner layer enhancement of MGC being higher than NMGC, although there are some reports that the inner layer of MGC is obviously enhanced [21]. We speculate that there may be three factors. Firstly, mucinous gastric carcinoma has been termed mucoid, colloid, or gelatinous carcinoma [21]. Histopathologically, mucinous carcinoma comprises large pools of extracellular mucin sparsely lined by columns of malignant cells, cords, and vessels. The higher proportion of myxoid matrix and enlarged extracellular space in mucinous carcinoma results in greater accumulation of contrast medium and lead to the higher enhancement of MGC. Secondly, mucinous gastric carcinoma is composed of a large amount of mucus and relatively small amount of tumor cell in the lesions. This seems to be the main reason for the low CT attenuation in the unenhanced phase. Based on the similar reasons, contrast medium is not easy to penetrate early. Therefore, the lesions were not enhanced obviously in the arterial phase. Finally, the contrast medium is slow to penetrate and usually exhibits persistent enhancement (in the portal venous and equilibrium phases) in mucinous adenocarcinoma, due to the presence of a large amount of mucus. However, because the incidence of MGC is low, further studies will be needed to corroborate these assumptions. The degree of enhancement of the middle or outer layer, the so-called “mucin pool”, in MGC was significantly lower than in NMGC. The majority of NMGC showed homogeneous enhancement, followed by heterogeneous enhancement and layered enhancement. Thus, mucinous gastric adenocarcinomas were characterized by layered enhancement due to the “mucin pool”, which was consistent with the research by Yan et al [11]. In this study, the enhancement patterns showed a certain correlation with tumor location and tumor staging. However, more cases are needed to confirm this correlation in further research.

The “mucin pool” is composed of extracellular mucus secreted by neoplastic cells, whose main component is high-molecular-weight mucin [22–24], which is divided into different mucin phenotypes [25]. Additionally, previous studies [26] have demonstrated that MGC cells can secrete large amounts of hydrolytic enzymes to hydrolyze the peripheral barrier structure, which is conducive to peripheral invasion and metastasis. These characteristics determine the pathological form and peripheral infiltration of MGC and may explain the layered enhancement and frequently advanced stage at the time of the initial diagnosis. The specific mechanism needs further study.

The incidence of calcification is extremely rare in NMGC [11] and relatively high in MGC. This study shows that the incidence of calcification is 33.3% in MGC, with different morphologies such as military, punctate and irregular calcifications. Calcification mostly occurs in the middle or outer layer, namely the “mucin pool”. This result may be due to alkaline mucins promoting calcific deposits in the “mucin pool” [11]. Therefore, calcification is another CT feature of MGC.

The data from our study revealed that the degree of enhancement of the middle or outer layer has better efficiency than other variables in distinguishing MGC and NMGC, especially in the arterial phase and portal venous phase. Layered enhancement has a high accuracy in identifying MGC and NMGC with high sensitivity and specificity, while calcification identifies them with a low sensitivity and high specificity. Therefore, the combination of layered enhancement and calcification is essential to distinguishing MGC from NMGC. Although obtained AUC was regarded as “fair” and did not reach “good” level, MDCT is still an important preoperative examination and some CT features such as layered enhancement and calcifications may be helpful in diagnosis of mucinous adenocarcinoma.

In clinical practice, the majority of patients with gastric cancer are often examined by contrast-enhanced CT before operation, radiotherapy and chemotherapy. Depending on layered enhancement and calcification, clinicians can determine the possibility of mucinous adenocarcinoma in their daily practice. It is very useful for old people with weak body, who cannot tolerate the endoscopic examination. In addition, clinicians can also estimate the bad prognosis of MGC, according to the clinicopathological characteristics, such as larger tumor size, more advanced tumor stage, and so on. The combination of clinicopathological characteristics and CT features in MGC can help clinicians formulate personalized therapeutic regimens and clinical nursing methods.

One disadvantage of this study is the small number of patients of advanced MGC with complete radiographic imaging, which limits the clear correlation between enhancement patterns and tumor location or tumor staging. Thus, further study with a larger sample size should be conducted to confirm our results.

In conclusion, MGC is a special histological type of gastric cancer and mostly occurs in older males with high malignancy. Compared to NMGC, MGC has a thicker gastric wall, larger tumor size, and is more frequently in an advanced stage when discovered. Layered enhancement and calcifications are characteristic CT features of patients with MGC.

## PATIENTS AND METHODS

### Patients

We retrospectively evaluated 62 patients with MGC and 104 patients with NMGC pathologically proven over the 10-year period spanning between 2003 and 2015 at Xinhua Hospital Affiliated Shanghai Jiaotong University School of Medicine, Shanghai, China. We included patients who underwent gastrectomy without preoperative chemotherapy or perioperative radiotherapy and had complete documentation including clinical characteristics, pathological findings, and operative procedure records. Inoperable or nonresected cases, such as exploratory laparotomy and gastrojejunal anastomosis, were excluded. Among all the enrolled patients, 30 patients with advanced MGC and 78 patients with advanced NMGC were examined by preoperative contrast-enhanced CT of the abdomen. This study was approved by the institutional review board, and informed consent was obtained from all patients before enrollment.

### CT Technique

All CT examinations were performed on multi-detector CT scanners (Somatom Definition, Siemens Medical Solutions, Germany). All patients fasted at least 8 hours prior to CT scanning. Each patient drank 750 ml water 1-2 hours before the examination and drank 250 ml water again 15 min before the examination to distend the stomach. The CT parameters were as follows: detector collimation (1 mm), pitch (0.9), gantry rotation (0.5), tube voltage (120 kVp), tube current (240 mAs), matrix (512×512), slice thickness (5 mm), and reconstruction interval (1 mm). The arterial phase, portal venous phase and equilibrium phase scan was obtained using a fixed 28 s, 60 s and 120 s equilibrium following intravenous injection of 100 ml of ionic contrast material at a rate of 3 ml/s using an automatic injector with a scan range from the diaphragm to the iliac crest. The range was expanded to the pelvis when distant metastasis was suspected.

### Clinicopathological Analysis

Clinicopathological variables were obtained from medical records and pathology reports, including patient gender, age, location of the tumor, diameter of the primary tumor, CEA, CA 19-9, macroscopic type, pathological depth of tumor invasion (pT), pathological number of metastatic lymph nodes (pN), pathological tumor stage of disease (pTNM), lymphatic invasion, venous invasion and neural invasion. Clinicopathological characteristics and macroscopic findings were analyzed in accordance with “Japanese classification of gastric carcinoma: 3rd English edition” by the Japanese Gastric Cancer Association [2]. Tumor size was recorded as the maximum diameter. The depth of infiltration was measured at the deepest point of penetration of the cancer cells. The macroscopic type was classified as type 0 (superficial), type 1 (mass), type 2 (ulcerative), type 3 (infiltrative ulcerative), type 4 (diffuse infiltrative) or type 5 (unclassifiable). Lymphatic, vascular, or perineural invasion was defined as the presence of permeation of the tumor in the lymphatic duct, vascular structure, or nerve, respectively, by microscopic examination [4]. The nodal classification was divided into four groups: pN0, no metastasis; pN1, one or two positive regional lymph nodes; pN2, 3-6 positive regional lymph nodes; and pN3, ≥7 positive regional lymph nodes.

### Image Analysis

The following items were analyzed for subjective evaluation: wall thickness of different portions, attenuation value of the inner layer, attenuation value of the middle or outer layer, degree and pattern of lesion enhancement, presence of calcification and ulceration. On enhanced CT scans, the inner layer corresponds to the mucosa and the muscular layer of the mucosa; the middle or outer layer corresponds to the submucosal layer and the muscle proper. For objective analysis, the CT attenuation values of the inner-layer and the middle or outer layer of the primary tumors were measured. As a reflection of the degree of enhancement, ΔCT value in arterial phase =attenuation value (HU) in arterial phase - attenuation value (HU) in unenhanced phase; the ΔCT value in the portal venous phase and in the equilibrium phase can be obtained similarly. The degree of enhancement of the tumor was based on CT imaging using HU attenuation, where “obvious enhancement” is defined as ΔCT ≥30 HU and “mild enhancement” as ΔCT <30 HU. The enhancement pattern of the lesion was categorized into three types: (A) homogeneous; (B) heterogeneous; or (C) layered (two- to three layered structures) and estimated in the portal venous phase on the axial CT images. The maximum thickness (mm) with wall thickening was also recorded. All CT images were reviewed by two board-certified abdominal radiologists (with 5 and 16 years of clinical experience in abdominal CT interpretation), and decisions were reached by consensus. All images were reviewed on a Picture Archiving and Communications System (PACS) workstation monitor (Syngo Multi-Modality Workplace, Version VE31A).

### Statistical Analysis

Statistical analysis was performed using the SPSS statistical package, version 19.0 (IBM, New York, NY), and P<0.05 was considered significant. The statistical methods used for the measurement data, presented as the mean±SD, were the t test or t’ test and qualitative data were evaluated using the χ^2^ test or Fisher's exact test. ROC curves were used to evaluate the variables in identifying MGC with NMGC.

## References

[1] Lauren P The two histological main types of gastric carcinoma: diffuse and so-called intestinal-type carcinoma. An attempt at a histo-clinical classification. Acta Pathol Microbiol Scand.

[2] Japanese Gastric Cancer Association Japanese classification of gastric carcinoma: 3rd English edition. Gastric Cancer.

[3] Bosman FT, Carneiro F, Hruban RH (2010). WHO classification of tumours of the digestive system.

[4] Hsu JT, Wang CW, Le PH, Wu RC, Chen TH, Chiang KC, Lin CJ, Yeh TS Clinicopathological characteristics and outcomes in stage I-III mucinous gastric adenocarcinoma: a retrospective study at a single medical center. World J Surg Oncol.

[5] Jass JR, Sobin LH, Watanabe H The World Health Organization's histologic classification of gastrointestinal tumors. A commentary on the second edition. Cancer.

[6] Sung CO, Lee SM, Choi JS, Kim KM, Choi MG, Noh JH, Sohn TS, Bae JM, Kim WH, Park CK, Kim S Tumor size predicts survival in mucinous gastric carcinoma. J Surg Oncol.

[7] Vernmark K, Albertsson M, Björnsson B, Gasslander T, Sandström P, Sun XF, Holmqvist A From palliative to curative treatment - stage IV mucinous adenocarcinoma, successfully treated with metronomic capecitabine in combination with Bevacizumab and surgery- a case report. BMC Cancer.

[8] Kim KH, Lee SH, Choi CW, Kim SJ, Choi CI, Kim DH, Jeon TY, Kim DH, Hwang SH Effect of extramucin pools in gastric cancer patients. Ann Surg Treat Res.

[9] Kunisaki C, Akiyama H, Nomura M, Matsuda G, Otsuka Y, Ono HA, Shimada H Clinicopathologic characteristics and surgical outcomes of mucinous gastric carcinoma. Ann Surg Oncol.

[10] Jian-Hui C, Shi-Rong C, Hui W, Jian-Bo X, Kai-Ming W, Si-le C, He YL Gastric mucinous cancer histology: clinicopathological characteristics and prognostic value. Gastroenterol Res Pract.

[11] Yan C, Zhu ZG, Yan M, Zhang H, Pan ZL, Chen J, Xiang M, Chen MM, Liu BY, Lin YZ Clinicopathological characteristics and computed tomography features of mucinous gastric carcinoma. J Int Med Res.

[12] Yu T, Wang X, Zhao Z, Liu F, Liu X, Zhao Y, Luo Y Prediction of T stage in gastric carcinoma by enhanced CT and oral contrast-enhanced ultrasonography. World J Surg Oncol.

[13] Jiang H, Zhang H, Tian L, Zhang X, Xue Y The difference in clinic-pathological features between signet ring cell carcinoma and gastric mucinous adenocarcinoma. Tumour Biol.

[14] Kim BS, Oh ST, Yook JH, Kim BS Signet ring cell type and other histologic types: differing clinical course and prognosis in T1 gastric cancer. Surgery.

[15] Choi MG, Sung CO, Noh JH, Kim KM, Sohn TS, Kim S, Bae JM Mucinous gastric cancer presents with more advanced tumor stage and weaker β-catenin expression than nonmucinous cancer. Ann Surg Oncol.

[16] Ryu SY, Kim HG, Lee JH, Kim DY Prognosis of early mucinous gastric carcinoma. Ann Surg Treat Res.

[17] Tang X, Zhang J, Che X, Lan Z, Chen Y, Wang C The clinicopathological features and long-term survival outcomes of mucinous gastric carcinoma: a consecutive series of 244 cases from a single institute. J Gastrointest Surg.

[18] Yin C, Li D, Sun Z, Zhang T, Xu Y, Wang Z, Xu H Clinicopathologic features and prognosis analysis of mucinous gastric carcinoma. Med Oncol.

[19] Solcia E, Luinetti O, Tava F, Klersy C, Grillo F, Pandolfo N, Fiocca R Identification of a lower grade muconodular subtype of gastric mucinous cancer. Virchows Arch.

[20] Zhang M, Zhu GY, Zhang HF, Gao HY, Han XF, Xue YW Clinicopathologic characteristics and prognosis of mucinous gastric carcinoma. J Surg Oncol.

[21] Park MS, Yu JS, Kim MJ, Yoon SW, Kim SH, Noh TW, Lee KH, Lee JT, Yoo HS, Kim KW Mucinous versus nonmucinous gastric carcinoma: differentiation with helical CT. Radiology.

[22] Chu PG, Chung L, Weiss LM, Lau SK Determining the site of origin of mucinous adenocarcinoma: an immunohistochemical study of 175 cases. Am J Surg Pathol.

[23] Bartley AN, Rashid A, Fournier KF, Abraham SC Neuroendocrine and mucinous differentiation in signet ring cell carcinoma of the stomach: evidence for a common cell of origin in composite tumors. Hum Pathol.

[24] Dilly AK, Lee YJ, Zeh HJ, Guo ZS, Bartlett DL, Choudry HA (2016). Targeting hypoxia-mediated mucin 2 production as a therapeutic strategy for mucinous tumors. Transl Res.

[25] Sasaki A, Kitadai Y, Ito M, Tanaka S, Yoshihara M, Haruma K, Chayama K Mucin phenotype and background mucosa of intramucosal differentiated-type adenocarcinoma of the stomach. Oncology.

[26] Choi JS, Kim MA, Lee HE, Lee HS, Kim WH Mucinous gastric carcinomas: clinicopathologic and molecular analyses. Cancer.

